# Bioenergetics of *Mycobacterium*: An Emerging Landscape for Drug Discovery

**DOI:** 10.3390/pathogens7010024

**Published:** 2018-02-23

**Authors:** Iram Khan Iqbal, Sapna Bajeli, Ajit Kumar Akela, Ashwani Kumar

**Affiliations:** Council of Scientific and Industrial Research, Institute of Microbial Technology, Chandigarh 160036, India; iramkhan@imtech.res.in (I.K.I.); sapnabajeli@imtech.res.in (S.B.); ajit.akela@imtech.res.in (A.K.A.)

**Keywords:** Mycobacterium tuberculosis, bioenergetics, oxidative phosphorylation, antimycobacterials, drugs, bedaquiline, Q203, SQ109, electron transport chain

## Abstract

*Mycobacterium tuberculosis* (Mtb) exhibits remarkable metabolic flexibility that enables it to survive a plethora of host environments during its life cycle. With the advent of bedaquiline for treatment of multidrug-resistant tuberculosis, oxidative phosphorylation has been validated as an important target and a vulnerable component of mycobacterial metabolism. Exploiting the dependence of Mtb on oxidative phosphorylation for energy production, several components of this pathway have been targeted for the development of new antimycobacterial agents. This includes targeting NADH dehydrogenase by phenothiazine derivatives, menaquinone biosynthesis by DG70 and other compounds, terminal oxidase by imidazopyridine amides and ATP synthase by diarylquinolines. Importantly, oxidative phosphorylation also plays a critical role in the survival of persisters. Thus, inhibitors of oxidative phosphorylation can synergize with frontline TB drugs to shorten the course of treatment. In this review, we discuss the oxidative phosphorylation pathway and development of its inhibitors in detail.

## 1. Introduction

Tuberculosis (TB) remains a leading cause of death worldwide, with an estimated 1.3 million mortalities in 2016. The synergy of human immunodeficiency virus (HIV)-TB co-infection has further aggravated this as a major public health problem. TB treatment is riddled with the use of multiple drugs for at least 6 months. Such lengthy treatment has resulted in the rise of multidrug resistant (MDR) and extremely drug resistant (XDR) strains of *Mycobacterium tuberculosis* (Mtb). Treatment of MDR TB requires administration of a multitude of second-line TB drugs for 18–24 months; this leads to cure rates of 60–70%. Of even more concern, cure rates for XDR-TB range from 40–50%. These low cure rates could be further confounded by other factors, such as co-infections and poor nutrition. Given these observations, new drugs with novel mechanisms of action are urgently required. Such innovative drugs could synergize with current treatment regimens to improve recovery rates and shorten treatment times. Extensive research efforts have been made in this direction, and the U.S. Food and Drug Administration has recently approved two new drugs. These drugs are bedaquiline (BDQ), a diarylquinoline ATP synthase inhibitor, and delamanid, a nitro-dihydro-imidazooxazole derivative that inhibits mycolic acid biosynthesis. Accelerated approval of these drugs has raised hope for a new regimen that could improve the outcome of treatment and reduce daily dose burden. 

The myth that targeting Mtb bioenergetics would be a poor therapeutic strategy due to the presence of parallel and alternative metabolic pathways has been dispelled by the discovery of BDQ. Given the success of this compound, several research groups have focused on targeting oxidative phosphorylation for the discovery of new drugs. It is important to note that, unlike many bacteria that can utilize substrate-level phosphorylation for ATP synthesis and growth, Mtb relies upon oxidative phosphorylation for its viability. During oxidative phosphorylation, electrons are harvested from central metabolic pathways, and then fed into the electron transport chain (ETC) through reduction of menaquinone. Later on, the quinones are re-oxidized by either the cytochrome *bc*_1_-*aa*_3_ complex or by the cytochrome *bd*-type terminal oxidase. The transport of electrons between membrane-bound primary dehydrogenase and terminal reductase is coupled to the generation of the proton motive force (PMF). The energy generated as PMF from this process is utilized by ATP synthase in the production of ATP. In this review, we have provided an overview of the machinery involved in oxidative phosphorylation and the development of inhibitors (listed in Table 1) targeting the components of oxidative phosphorylation.

## 2. Feeding the Electrons into the Electron Transport Chain: Reduction of Menaquinone

During the infection cycle, Mtb cells harvest reductive energy from host-derived carbohydrates and fatty acids using NADH/NAD^+^ and/or FADH_2_/FAD redox pairs [1,2]. NAD^+^ acts as the primary electron sink and is converted into NADH, which feeds electrons into the ETC through the NADH dehydrogenase complex. Besides NADH dehydrogenase, a number of other primary dehydrogenases pump electrons directly into the ETC, such as succinate dehydrogenase (SDH), proline dehydrogenase, and L-lactate cytochrome *c* oxidoreductase. In this review, we have primarily focused on NADH dehydrogenase and SDH.

### 2.1. NADH/Menaquinone Oxidoreductase

NADH/menaquinone oxidoreductases are the primary port of entry for electrons into the ETC. There are three types of these oxidoreductases in bacteria: (i) the highly-complex, multi-subunit-proton-pumping type I NADH dehydrogenase (NDH-1), (ii) a simple, single subunit flavoenzyme-non-proton-pumping type II NADH dehydrogenase (NDH-2), and (iii) a sodium-pumping NADH dehydrogenase (NQR) that is unique to bacteria [3]. In 2005, using a genome mining approach, Weinstein et al. identified NDH-1 and NDH-2 in Mtb [4]. NDH-1 is encoded by the *NuoABCDEFGHIJKLMN* operon and is predicted to be the energy efficient NADH dehydrogenase that translocates protons, while transferring electrons to menaquinone to generate PMF. Interestingly, genes encoding NDH-1 are not essential in Mtb [5], as exemplified by their loss from *Mycobacterium leprae* through genome reductive evolution [4]. NDH-1 is downregulated by acidic pH [6], nutrient starvation [7], and in murine lungs [8]. This expression profile suggests that NDH-1 plays a minor role in Mtb respiration, and thus, is not a good target for the development of antimycobacterials. This view is further strengthened by the observation that rotenone (an NDH-1 inhibitor) does not kill hypoxia-adapted Mtb cells [9]. However, recently developed reporter strains for imaging cellular NADH/NAD^+^ levels [10] has demonstrated that exposure of aerobic Mtb cultures to rotenone leads to accumulation of cellular NADH levels, suggesting a role for NDH-1 in the ETC during aerobic conditions. Furthermore, infection of macrophages with an Mtb strain that has a deletion of *nuoG* (which encodes an NDH-1 subunit) accelerates their apoptotic death. Moreover, this mutant has reduced survival in a murine model of TB infection [11].

The non-proton pumping NDH-2 isoforms are encoded by *ndh* (Rv1854c) and *ndhA* (Rv0392c). Both the two isoforms were found to be functional in biochemical assays. NDH-2 has a stringent catalytic function, thereby minimizing leakage of electrons to oxygen during their transfer from NADH to menaquinone. Importantly, Mtb NDH-2 utilizes only NADH, in contrast to NDH-2 of *Saccharomyces* and *Corynebacterium*, which uses NAD(P)H [12]. Mtb NDH-2 transfers electrons to the quinone pool by a ping-pong mechanism [13]. Sena et al. demonstrated that NDH-2 has two distinct substrate binding sites (i.e., NADH and quinone). NDH-2 first interacts with NADH to receive electrons, and then releases NAD^+^ before interacting with the quinone [14]. It is assumed that the reduction of quinone/menaquinone is a rate-limiting step in the overall reaction [15]. Both the isoforms of NDH-2 are conserved in slow-growing mycobacterial species, with the exception of *M. leprae*, in which *ndhA* is absent [4]. *ndh* is essential for Mtb survival [5], whereas *ndhA* disruption using transposon mutagenesis is tolerated [16]. Importantly, disruption of NDH-2 through inhibitors, such as phenothiazine analogues, leads to mycobacterial death in aerobic cultures and in animals [4]. In agreement with these findings, the NDH-2 inhibitor thioridazine (TZ) kills Mtb that are in a hypoxia-induced non-replicating state [9]. *ndh* is also induced upon Mtb infection of macrophages [17]. Interestingly, mammalian mitochondrion lacks an NDH-2 orthologue, which provides a significant therapeutic window. Earlier studies have also validated NDH-2 as a target to control Plasmodium growth [18].

### 2.2. Inhibitors of NADH Dehydrogenase

The above cited literature strongly suggests that NDH-2 is an attractive target whose inhibition would elicit a choke point in the mycobacterial respiratory chain. In an effort to discover additional respiratory inhibitors, Harvey and colleagues found that phenothiazine analogues have antimycobacterial activity and demonstrated that these compounds inhibit NDH-2 [4]. They also demonstrated that classical inhibitors of NDH-1 (piericidinA, rotenone, and pyridaben) do not inhibit Mtb respiration. Phenothiazines were also effective against MDR strains, suggesting a new mode of action [19,20]. Trifluoperazine (TPZ), a phenothiazine analogue, can effectively inhibit Mtb growth, and synergizes with rifampicin (RIF) to kill intracellular Mtb residing in macrophages [21,22]. A recent report indicates that TPZ significantly inhibits ATP synthesis in *M. leprae* [23]. Phenothiazines also inhibit efflux pumps and calcium-binding protein. Similar antimycobacterial activity (both in drug-sensitive and drug-resistant strains) was observed with the NDH-2 specific inhibitor, thioridazine (TZ), in Mtb-infected mice [24]. TZ has been used for 60 years to control psychosis and is considered therapeutically safe. The drug targets NDH-2 to block the ETC of mycobacteria and is effective against latent TB [13,25]. Given the critical role of NDH-2 and the potent activity of TPZ and TZ against Mtb cells, high throughput screening (HTS) was performed with 11,000 compounds rationally selected from a commercial library (Biofocus, DPI). This effort resulted in the identification of lead compound 42a (MTC420) that had a minimum inhibitory concentration (MIC) in the nanomolar range. This lead compound is capable of killing drug-sensitive and -resistant strains of Mtb in aerobic and hypoxic cultures. Furthermore, 42a also has favorable pharmacokinetic and toxicological profiles [26]. 

Clofazimine (CFZ) is derived from phenazines, and has potent anti-tuberculosis activity [27]. The drug effectively kills *M. leprae*, and is therefore used for the treatment of leprosy [28]. The main side effect of this drug is skin pigmentation. Recently developed CFZ derivatives namely, B746 and B4157, have increased anti-mycobacterial activity and elicit less pigmentation, and are therefore suitable for testing for treatment of mycobacterial infection in animal models [29,30]. An eminent study from the Harvey group demonstrated that the bactericidal action of CFZ depends upon its reduction by NDH-2. The reduced form of CFZ is highly unstable, and spontaneously reacts with oxygen to generate reactive oxygen species (ROS) that leads to the killing of mycobacterial cells [31]. This cycle of CFZ-mediated ROS generation continues under aerobic conditions. Importantly, the NDH-1 present in mitochondrial and bacterial respiratory chains does not reduce CFZ. Therefore, Gram-negative bacteria, including *Escherichia coli*, *Pseudomonas denitrificans*, and *P. aeruginosa* do not produce bactericidal ROS upon exposure to CFZ [32,33]. CFZ is also active against MDR TB strains, is metabolized slowly, and is associated with a low frequency of resistance [34]. CFZ synergizes with BDQ and Q203 to rapidly kill Mtb in vitro and inside macrophages [35]. 

Since NDH-2 is a respiratory choke point of Mtb, AstraZeneca (India) performed an HTS against Mtb NDH-2 with 100,000 compounds. This effort identified quinolinyl pyrimidines as a new class of NDH-2 inhibitors. A good correlation was observed between enzyme inhibition (nanomolar) and anti-mycobacterial activity (micromolar) of quinolinyl pyrimidines [36]. These compounds are non-toxic and have desirable absorption, distribution, metabolism, and excretion (ADME) profiles, making them suitable for further development. In line with these findings, compounds belonging to 7-phenyl benzoxaborole series exhibited potent anti-Mtb activity [37].

### 2.3. Succinate Dehydrogenase 

The succinate dehydrogenase (SDH) or succinate/menaquinone oxidoreductase forms complex II of the respiratory chain. Importantly, this enzyme complex is also an integral part of the tricarboxylic acid (TCA) cycle. SDH links the respiratory chain with central metabolism [38]. The enzyme oxidizes succinate to fumarate in the cytoplasm, and during this process, it simultaneously reduces menaquinone to menaquinol in the membrane. This enzyme complex is comprised of four subunits: SdhA, SdhB, SdhC, and SdhD. SdhA is a flavoprotein (flavin is bound here covalently, and not as a cofactor) that catalyzes the conversion of succinate into fumarate. SdhB possesses three Fe−S clusters that facilitate the transfer of electrons from succinate to menaquinone. The SdhC and SdhD subunits anchor SdhA and SdhB to the membrane, facilitating the transfer of electrons from succinate to menaquinone using haem. Notably, fumarate reductase (FRD) is a paralogue of SDH that can catalyze the reverse reaction. FRD (encoded by the FrdA-Rv1556 operon) is primarily functional in anaerobic conditions. Mtb utilizes menaquinone (MQH2 E′_0_ = −74 mV) to reduce fumarate in the last step of the anaerobic ETC [39,40,41]. Interestingly, Mtb possesses two isoforms of SDH; Sdh1 (encoded by Rv0247c–Rv0249c) and Sdh2 (encoded by Rv3316–Rv3319). It is crucial to note that under hypoxic conditions, Mtb cells utilize the reverse TCA cycle to accumulate and secrete succinate into the culture medium [36,42]. The excretion of succinate has been associated with the upregulation of genes involved in the reverse TCA cycle. Addition of succinate to the culture medium resulted in membrane potential depolarization and cell death, suggesting that secretion of succinate is critical for surviving hypoxic stress [42,43]. A role for SDH in this adaption had been predicted, since inhibition of SDH function using 3-nitropropionate (3NP) results in decreased survival of Mtb [42]. Individual Sdh1 and Sdh2 deletion mutants were generated in order to dissect the role of both Sdh1 and Sdh2 during hypoxic adaptation. This revealed that survival of the Sdh1 mutant was impaired in stationary phase, while the survival of the Sdh2 mutant was not affected [44]. The function of Sdh1 as the catalytic center during aerobic conditions was demonstrated through the use of stable isotope labelling and mass spectroscopy [44]. Sdh2 was not required for this activity during aerobic conditions. Furthermore, depletion of Sdh1 resulted in increased levels of the reduced lipogenic electron carrier, menaquinol, which was associated with an increased rate of respiration, suggesting that SDH is a regulator of respiration [44]. Importantly, survival of the Sdh1 mutant was compromised in the Mtb-infected C3HeB/FeJ mouse model system, which produces lesions similar to the hypoxic granulomas found in humans upon Mtb infection [44]. These observations suggest that inhibition of SDH can help in the elimination of hypoxia-induced persisters in the infected lungs. Sdh1 is, thus, a potential drug target, however, stringent selectivity will be required to avoid a general inhibition of mitochondrial respiration.

### 2.4. Menaquinone Biosynthesis and Inhibitors of Menaquinone Biosynthesis Pathway in Mtb

**Figure 1:**
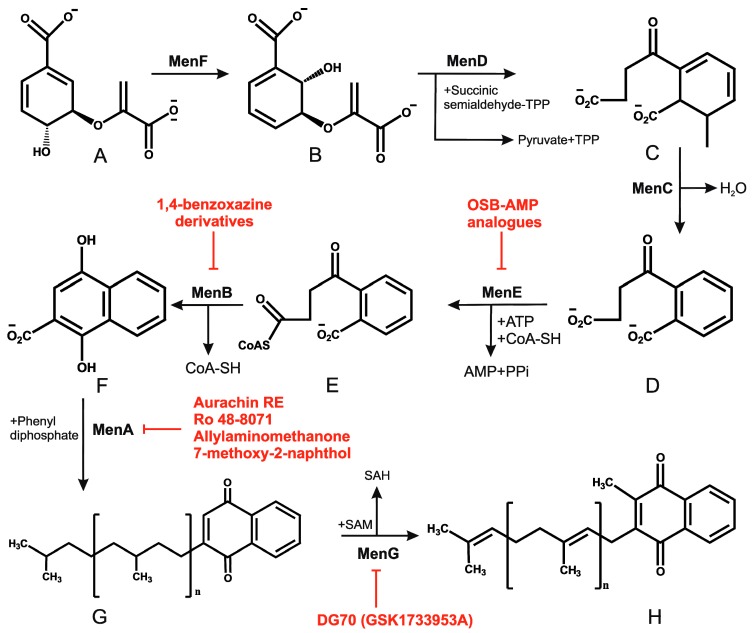
Mycobacterial menaquinone biosynthesis pathway and its inhibitors. (**A**–**H**) represent chorismate, isochorismate, 2-succinyl-6-hydroxy-2,4-cyclohexadiene-1-carboxylate, *o*-succinylbenzoate, *o*-succinylbenzoyl-CoA, 1,4-dihydroxy-2-naphthoyl CoA, demethylmenaquinone, and menaquinone respectively. Drugs that target the menaquinone biosynthesis enzymes are shown by red flathead arrows.

Ubiquinone (Q) and menaquinone (MK) are the primary lipid-soluble electron carriers that transport electrons in the ETC [1]. In most Gram-positive bacteria and in mycobacteria, MK is the primary electron carrier, while Gram-negative bacteria possess both Q and MK [45]. Mycobacterial species, including Mtb, possess MK-9(H_2_) (hereafter MK) [46,47]. Besides playing the role as an electron carrier, MK assists in the proper folding of secreted proteins through vitamin K epoxide reductase or VKOR [48]. The ratio of oxidized to reduced MK is sensed by the DosRST system to control mycobacterial metabolism [49,50]. Since MK is the only electron carrier in mycobacteria, and because humans do not synthesize this vitamin, it is believed to be an excellent target for the development of drugs against mycobacterial pathogens. MK is synthesized in bacteria through a classical pathway and a second, alternate pathway. Both pathways begin with a chorismate molecule, which is then metabolized to a different MK precursor, depending on whether the classical or alternate pathway is engaged. Although the alternate pathway is operational in evolutionarily related *Streptomyces*, it has not been reported in mycobacteria. In regard to the classical pathway, chorismate is converted into MK through a series of reactions carried out by enzymes encoded by the MenFDHCEBA cluster [5], as summarized in Figure 1. Some of these enzymes are essential for mycobacterial growth [5,51] suggesting that MK biosynthesis is a valid drug target. 

Since cholesterol metabolism is important for mycobacterial survival within the host, the effect of exposure of Ro 48-8071 (an inhibitor of cholesterol biosynthesis) on mycobacterial growth was analyzed. This compound killed *Mycobacterium bovis* Bacillus Calmette Guerin (BCG) cells at low micromolar concentrations [52]. Further metabolic labelling experiments identified MK biosynthesis to be the target of Ro 48-8071. These findings were supported by the observation that Ro 48-8071 was capable of reducing oxygen consumption during growth. Further scrutiny led to the identification of MenA (Rv0534c) as the target of Ro 48-8071. The same group utilized a rational drug design approach to target MenA. They screened around 100 molecules, and established that an allylaminomethonone class of compounds are potent inhibitors of Mtb strains, including those which are drug-resistant [53]. This class of MenA inhibitors was also highly effective against hypoxia-induced drug-tolerant persisters [54]. Interestingly, aurachin RE, a new quinoline antibiotic isolated from *Rhodococcus erythropolis* JCM 6824 is highly effective against Gram-positive bacteria [55]. The aurachin RE molecules were modified to specifically target MenA, and validated as MenA inhibitors in functional in vitro assays. These compounds were low micromolar inhibitors of aerobic growth and hypoxic survival of Mtb [56]. Additionally, 7-methoxy-2-naphthol-based molecules have been utilized as lead structures for the development of non-traditional inhibitors of MenA of Mtb [57]. These efforts led to the development of bicyclic inhibitors that exert potent growth-suppressing activity against Mtb [57]

High-density phenotypic profiling has identified *menB* (Rv0548c) as essential for mycobacterial growth [58]. MenB catalyzes the formation of 1,4-dihydroxy-2-naphthoyl-CoA from *o*-succinylbenzoyl CoA through a Claisen condensation reaction. Li and coworkers performed an HTS with more than 100,000 compounds, which identified a few hundred leads. Subsequent elaboration yielded multiple 1,4-benzoxazine compounds that were potent inhibitors of MenB activity, and consequently blocked mycobacterial growth [59]. These leads were then utilized for the development of 4-oxo-4-chlorophenylbutenoyl methyl ester derivatives with potent mycobactericidal activity against replicating and non-replicating Mtb [60].

MenE (Rv0542c) is the fifth enzyme in the MK biosynthesis pathway, and catalyzes the formation of OSB-CoA from O-succinyl-1-benzoate (OSB) through two mechanistic steps. The first is the ATP-dependent adenylation of OSB, and the second step is thioesterification with CoA. Given the structural and functional similarity of MenE with adenylate-forming enzymes, it is a promising target of acyl-AMP intermediates. Considering the mechanistic details, Tan and coworkers employed OSB-AMP analogues to inhibit MenE in Mtb [61]. The IC_50_ of OSB-AMP analogues MeOSB-AMS, MeOSB-AMSN, and MeOSB-AVSN for MenE are in the low micromolar range [61]. Further development of these analogues resulted in the identification of OSB-AMS with an IC_50_ for MenE in the nanomolar range; these compounds are competitive inhibitors of the binding of ATP and OSB [62]. Paradoxically, these inhibitors had poor mycobacterial growth inhibitory activity, perhaps due to their instability, and the OSB-AMP analogues were, therefore, further modified. This resulted in the development of OSB-AMS. Importantly, the difluoroindanediol analogue 11 of OSB-AMS was an effective mycobactericidal compound [63]. 

In an effort to identify inhibitors of oxidative phosphorylation, Alland and coworkers created a cell-based screen [64]. In this screen, the *mWasabi* reporter was fused to the promoter of the *cydAB* operon (coding for part of cytochrome *bd* oxidase) to monitor inhibition of respiration in the presence of 168 known inhibitors of mycobacterial growth. This led to the identification of DG70 as an inhibitor of bacterial respiration. DG70 is a biphenyl benzamide that kills both drug-sensitive and -resistant Mtb cells [64]. Interestingly, this novel inhibitor is highly specific against Mtb, and does not kill non-tuberculous mycobacteria (NTMs), Gram-positive, Gram-negative, or ESKAPE (*Enterococcus faecium*, *Staphylococcus aureus*, *Klebsiella pneumonia*, *Acinetobacter baumannii*, *P. aeruginosa*, and *Enterobacter*) pathogens. Sequencing of two DG70-resistant spontaneous mutant strains suggested the presence of a unique single nucleotide polymorphism (SNP) in *menG*. MenG (Rv0558, also known as MenH) catalyzes the conversion of demethylmenaquinol to menaquinol. Importantly MK4, an analogue of MK9, rescued the DG70-mediated killing of Mtb cells, thus validating that MK biosynthesis is the target of DG70. Importantly, DG70 synergizes with BDQ, isoniazid (INH), and PA824 to kill Mtb [64]. In summary, all these observations indicate that small molecule targeting of MK biosynthesis (summarized in Figure 1) is a valid therapeutic strategy.

## 3. Oxidation of Menaquinone: A Tale of Two Terminal Oxidases

Mycobacteria possess a branched ETC. During aerobic respiration, electrons are fed into the menaquinone_(reduced/oxidized)_ pool of respiratory chains. Electrons are then transferred to oxygen through two branches, each of which possesses a terminal oxidase. One is cytochrome *bd*-type menaquinol oxidase (encoded by *cydABDC*), wherein electrons are transferred directly from MK (reduced) to oxygen. The other branch runs through a supercomplex of menaquinol-cytochrome *c* oxidoreductase or the *bc*_1_ complex and *aa*_3_ type cytochrome oxidase [1,65,66,67]. The *bc*_1_-*aa*_3_ branch pumps protons out of the cell during transfer of electrons to oxygen, whereas the *bd*-type oxidase does not pump protons, but has a higher affinity for oxygen [68]. It is assumed that similar to *E. coli*, the two terminal oxidases of mycobacteria have different affinities for oxygen; however, this awaits experimental validation. 

### 3.1. bc_1_-aa_3_ Pathway

The *bc*_1_ complex is a key component of the *bc*_1_-*aa*_3_ pathway, and is required for the bulk of the electron transfer to oxygen during normoxia [69]. *bc*_1_ is encoded by the *qcrCAB* operon in Mtb. It consists of redox groups comprising a 2Fe/2S centre, located on a Rieske protein (QcrA), two *b*-type haems (low and high potential) located on a single polypeptide (QcrB), and the haem of cytochrome *c*_1_ (QcrC). QcrA of Mtb has three transmembrane helices and characteristic sequence motifs (CSHLGC and CPCH) of 2Fe–2S Rieske iron-sulfur proteins, QcrB has a 120 amino acid extension at the C-terminus, and the QcrC subunit consists of two haem binding motifs (CVSCH and CASCH) for *c*-type cytochromes, suggesting a covalent di-haem (*bcc*) configuration [70]. According to Q-cycle model, oxidation of quinol molecules occurs at the interface of cytochrome *b* and the 2Fe–2S cluster carrying domain of the Rieske protein, which forms the catalytic center P at the positively charged membrane side of the enzyme [71]. Each quinol molecule oxidizes at the center P, liberating two electrons that move towards different acceptors. One electron proceeds to the iron-sulfur Rieske protein, and is then transferred to cytochrome *c*, while the other moves to another quinone-binding site on the opposite side of the membrane through haem molecules of variable redox potentials. Complex III releases four protons for every two electrons transferred from menaquinol to cytochrome *c* into the periplasmic side of the membrane [69]. 

The Mtb *aa_3_*-type cytochrome *c* oxidase is comprised of four subunits: CtaB (a cytochrome *c* oxidase assembly factor), CtaD (cytochrome *c* oxidase subunit I containing haem *a*, *a_3_*, and copper_B_ (Cu_B_), CtaC (cytochrome *c* oxidase subunit II containing copper_A_ (Cu_A_) and CtaE (subunit III). The *aa*_3_ cytochrome *c* oxidase of Mtb is encoded by *ctaBCDE*, which is dispersed over different locations of the Mtb genome [72]. Interestingly, the *ctaE* gene is located immediately upstream of the *qcrCAB* operon. The putative fourth subunit is encoded by *ctaF* (Rv2199c). This subunit is co-purified with other subunits in *Corynebacterium*. Importantly, the deletion mutants display the phenotype similar to CtaC mutant [73]. Four protons are taken up, while two protons are released into the periplasm for every two electrons passing through complex IV [72]. There are three *ctaD* alleles in *Mycobacterium smegmatis*, while only one in Mtb, indicating the presence of multiple isoforms of cytochrome *c* oxidase in *M. smegmatis* [65]. The proton-pumping type (and energetically more efficient) *bc*_1_-*aa*_3_ branch of ETC is essential for mycobacteria, as its deletion by homologous recombination is lethal [66]. This observation indicates that the *bc*_1_-*aa*_3_ branch of ETC is an important drug target. 

Unlike *E. coli*, which utilizes membrane-soluble cytochrome C for transferring electrons between *bc*_1_ complex and *aa*_3_ cytochrome *c* oxidase, *Mycobacterium*, *Corynebacterium glutamicum*, and *Rhodococcus* do not possess cytochrome C [70,74,75]. Importantly, in *C. glutamicum*, the QcrC contains an extra binding site for another haem *c* that may function as a merged cytochrome C [76]. Moreover, subunit II of the oxidase contains an additional 30 amino acids that could participate in the direct interaction between complexes *bc*_1_ and *aa*_3_, to create a respiratory “supercomplex” [66], obviating the requirement for free cytochrome *c* [74,76]. Indeed, in *C. glutamicum*, *bc*_1_ and *aa*_3_ are isolated as a complex that is resistant to detergent treatment [73]. A similar scenario was predicted for mycobacteria [74], and was validated following the isolation of a detergent-resistant supercomplex in *M. smegmatis* [77]. The interaction between the *bc*_1_ and *aa*_3_ complexes is guided by hydrophobic interactions, while ionic interactions facilitate the alignment between the two complexes for efficient electron transfer from menaquinol to oxygen [77]. Recently, overexpression of the Mtb complex III in *M. smegmatis* was shown to yield a functional, stable, hybrid supercomplex in the presence of dodecyl maltoside detergent [78]. These observations strongly suggest that, in mycobacterial cells, the *bc*_1_ complex and *aa*_3_ cytochrome *c* oxidase interact with each other to facilitate the flow of electrons from MK to oxygen, without the requirement of cytochrome C. 

### 3.2. Cytochrome bd-Oxidase

Cytochrome *bd*-type menaquinol oxidase (*bd*-oxidase) in Mtb is non-proton pumping, and is therefore a less energetically efficient terminal oxidase. *E. coli bd*-oxidase is the prototypical member of this enzyme class and is encoded by two separate operons, *cydAB* and *cydDC*. This is contrary to Mtb, in which all the genes are transcribed from a single *cydABDC* operon. *cydAB* encodes the functional cytochromes, while the products of *cydDC* contribute to cytochrome *bd* assembly [79]. Furthermore, in *E. coli* mutants of *cydDC*, the periplasmic space is more oxidized than in the wild type bacteria [79]. The function of *cydDC* in mycobacteria is largely unknown, but it plays an important role in mycobacterial persistence in vivo. *cydDC* mutation reduces the ability of Mtb to survive the transition from acute to chronic infection in mice [12]. Another report suggests that *cydDC* supports mycobacterial persistence in INH-treated mice [13]. Compared to their wild type counterparts, cytochrome *bd* mutants of *E. coli* are sensitive to stress induced by temperature alterations, nitric oxide, H_2_O_2_, and iron (III) chelators. Additionally, they are unable to resume growth upon entering into the stationary phase [80,81,82,83]. Importantly, the growth of *cydA* mutant *M. smegmatis* is normal at ambient oxygen levels, but is severely impaired during hypoxia (0.5–1% air saturation). These mutants are also sensitive to cyanide, and are outcompeted when co-cultured with wild type *M. smegmatis* in its presence [65]. In *E. coli*, *bd*-oxidase has a higher affinity for oxygen, and is induced in response to low oxygen tension [84]. Although the affinity of the Mtb *bd*-oxidase for oxygen has not been determined, its expression is also induced during hypoxia [65]. The expression of *cydAB* is regulated by a SenX3-RegX3 two-component system [85], which also acts as an oxygen-controlled replication switch in Mtb [86]. It was recently demonstrated that Mtb *cydAB* mutants are sensitive to H_2_O_2_ and antibiotic stress [87]. Importantly, drugs inhibiting mycobacterial respiration enhance the expression of *cydAB* [88]. Interestingly in *M. smegmatis*, inactivation of *bc*_1_ complex results in the upregulation of the *bd*-type terminal oxidase; however, the *bc*_1_ complex does not compensate for the loss of the *bd*-type oxidase [87]. Genetic inactivation of cytochrome *bd*-oxidase in various pathogenic microorganisms like *Shigella flexneri*, *Brucella abortus*, and *Salmonella enterica* serovar *Typhimurium*, impairs their intracellular survival and virulence [89,90,91].

### 3.3. Supercomplex Inhibitors

The essentiality of Mtb cytochrome *bc_1_*, along with the large differences in structure and function from its mammalian counterpart, makes it a good target for therapeutic intervention [78]. A variety of antibiotics and chemical compounds have long been known to inhibit the *bc*_1_-*aa*_3_ complex. The aurachin class of compounds contains quinone analogues, which are reported to inhibit a variety of cytochrome oxidases [56,92,93]. Aurachin D is well-known among this class of compounds that inhibit *E. coli* cytochrome *bd*-oxidase [92]. It inhibits oxygen consumption in *M. smegmatis* in a dose-dependent manner [87]. These observations demonstrate the importance of cytochrome *bd* inhibitors as drug molecules. Optimized derivatives of aurachin D, with better ability to penetrate the mycobacterial cell wall, have a great potential as a new class of antitubercular drugs [87].

Similar to aurachin D, myxothiazol-based compounds, which are isolated from *Myxococcus fulvus*, target mitochondrial cytochrome *b*. Myxothiazol interacts both with cytochrome *b* and an iron-sulfur protein of the complex; this displaces quinone from the high-affinity binding site of the iron-sulfur protein [94]. Importantly, myxothiazol inhibits the growth of *Mycobacterium* sp. GBF 3 at an MIC value of 6.3 μg/mL [95], indicating that respiratory inhibitors can successfully target mycobacterial species. Interestingly, myxothiazol does not kill Gram-negative or -positive bacteria, but does exhibit strong antifungal activity [95]. A few other antifungal antibiotics, such as mucidin (from *Basidiomycetes Oudemansiella mucida*) and strobilurin A (from *Strobilurin stenacellus*) also inhibit the *bc*_1_ complex by binding to the same site as myxothiazol [96]. However, antimycin A (another antibiotic) binds to a different location, inhibiting the oxidation of cytochrome *b* subunit [97]. A screen in search of new antibiotics identified a novel compound, stigmatellin, from *Stigmatella aurantiaca* strain Sg a15, that showed activity against various Gram-positive bacteria, yeasts, and filamentous fungi [98]. Subsequently, it was found to block mitochondrial electron transport by inhibiting the cytochrome *bc*_1_ complex as effectively as antimycin A and myxothiazol [99].

The capability of a number of small molecules to specifically target energy production in Mtb has validated oxidative phosphorylation as a viable drug target. Since energy production through oxidative phosphorylation plays an important role in Mtb survival during hypoxia-induced non-replicating persistence [9,100], blockade of oxidative phosphorylation could be pursued, therapeutically, to kill persisters. A number of small molecule inhibitors that specifically target the *bc*_1_-*aa*_3_ complex have been identified. Several lines of evidence suggest that these inhibitors, which are structural analogues of quinone, target the quinone binding catalytic domains of the *bc*_1_ complex [101]; prominent examples are the imidazo[1,2-a]pyridines (IPs). Using HTS, IPs were identified as potent inhibitors of Mtb and BCG [101]. Importantly, four IP inhibitors were shown to specifically target Mtb strains and BCG, but were not able to kill a number of other Gram-positive and Gram-negative bacteria, or human cell lines, including HepG2 and Neuro2A. Whole genome sequencing of the spontaneous resistant mutants established that these IPs targeted QcrB. These findings were further supported by induction of drug resistance upon overexpression of *qcrB* [101]. HTS of 100,997 compounds led to the discovery of phenoxyalkylbenzimidazole (PAB) class of compounds showing activity against Mtb with MICs in the nanomolar range, and low cytotoxicity against eukaryotic cells [102,103]. A recent study identified the probable target of PABS to be the QcrB subunit of cytochrome *bc*_1_ oxidase, further confirming it to be an important drug target [104].

**Figure 2:**
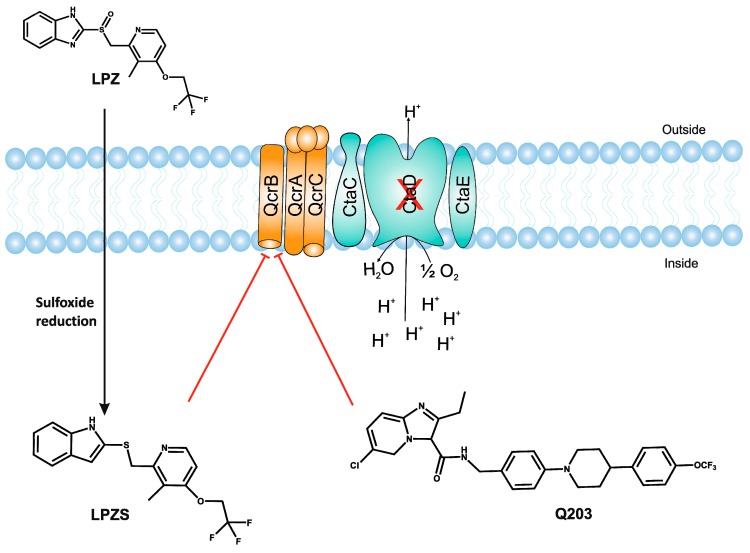
Inhibition of cytochrome *bc_1_* by LPZ and Q203. The sulfoxide reduction of LPZ converts it to active LPZS, which can bind QcrB of cytochrome *bc*_1_ complex. Q203, an imidazopyridine amide, also targets the QcrB subunit of cytochrome *bc*_1_ complex. Inhibition of QcrB forces the mycobacteria to use energetically less efficient cytochrome *bd* oxidase, a decrease in proton motive force (PMF) and ATP levels. Red flathead arrows indicate binding with subunit and inhibition of cytochrome *bc*_1_ complex.

An independent screen of more than 100,000 compounds to identify inhibitors of macrophage-resident bacterial growth led to the isolation of two highly active IP compounds with less toxicity than previous molecules of the same class. Further lead optimization resulted in the development of IP Q203, which is specifically active against Mtb (MIC_50_ of 2.7 nM in broth cultures and 0.28 nM for intracellular bacteria) [105]. Furthermore, whole genome sequencing of spontaneously resistant mutants revealed the target of Q203 to be the cytochrome bc_1_ complex (*qcrB*) (as depicted in Figure 2). Mutation of Thr313 to either alanine or isoleucine was specifically involved in Q203 resistance [34]. Importantly, this compound was highly active against MDR and XDR clinical isolates [106]. This leading drug candidate has recently progressed to Phase I clinical trials (trial identifier: NCT02858973) under a U.S. FDA Investigational New Drug application. Notably, Q203 is synthesized through a one-pot reaction of 2-aminopyridines or 2-(or 4-)aminopyrimidines, respectively, with 1,2-bis(benzotriazolyl)-1,2-(dialkylamino)-ethanes [107]. These studies were followed by derivatization of imidazo[1,2-a]pyridine 3 carboxyamides into imidazo[1,2-b]thiazole-5-carboxyamides. Structure–activity relationship (SAR)-based assays were utilized for optimizing the imidazo[1,2-b]thiazole-5-carboxyamides to yield three lead compounds that have potent antitubercular activity at low nanomolar concentrations. These compounds specifically inhibit QcrB, and thus, block the growth of replicating Mtb and intracellular mycobacteria, and display very low toxicity [108]. These compounds were also active against large numbers of NTMs, including *Mycobacterium avium* in murine lungs [108,109]. In another parallel drug discovery effort, an HTS campaign by Novartis Institute for Tropical Diseases identified several potential hit molecules with potent activity against Mtb [110]. However, the presence of ester linkages raised concerns over their metabolic instability [111]. Several compounds were thus optimized for metabolic stability through pharmacokinetic studies in the mouse. These studies led to the development of pyrrolo[3,4-c]pyridine-1,3(2H)-diones having an MIC_90_ in the micromolar range [111]. These compounds are hyperactive against cytochrome *bd*-oxidase mutants, suggesting that they target the *bc*_1_-*aa*_3_ complex of the respiratory chain. The target for these molecules was identified as *qcrB*, and a point mutation (Ala317Thr) in *qcrB* results in resistance to these compounds [111]. Importantly, the target site is identical to that bound by Q203, suggesting that this site may be promiscuous for molecules targeting the *bc*_1_ complex.

Interestingly, in an effort to build a strong pipeline for antimycobacterial drugs, a pool of 1280 FDA approved drugs was screened for growth inhibition of Mtb residing in MRC-5 lung fibroblasts. This screen resulted revealed that the gastric proton pump inhibitor lansoprazole (LPZ, Prevacid) exerted potent activity against Mtb. Importantly, other proton pump inhibitors, such as omeprazole and pantoprazole, were not able to inhibit Mtb growth. It was further demonstrated that LPZ is rapidly converted to the potent antimycobacterial agent, lansoprazole sulfide (LPZS). Thus, LPZ acts as a prodrug that is converted into LPZS within the host cell. As the conversion of LPZ into LPZS is inefficient, a 22-fold higher drug dose was required to kill Mtb cells in broth cultures. LPZS selectively kills Mtb; it is not toxic to closely related NTMs or Gram-positive and-negative bacteria [112]. Target identification studies revealed that LPZS targets the cytochrome *bc*_1_ complex (as depicted in Figure 2) of the respiratory chain, and conversion of leucine-176 to proline in *qcrB* confers drug resistance. Furthermore, superimposition of the mutant mycobacterial protein structure onto the published QcrB protein from *R. sphaeroides* [113] revealed that both L176P (a mutation leading to LPZS resistance) and T313A (which confers IPA resistance) were localized to the same site, i.e., the P site, at which ubiquinol oxidation occurs. However, L176P mutants remained susceptible to various IPA, whereas T313A mutants were susceptible to LPZS, revealing different binding mechanisms for each compound [112]. There are conflicting reports on the oral bioavailability of this drug. For example, oral administration of the drug is reported to significantly reduce mycobacterial burden [112], whereas others have shown that intraperitoneal injection of LPZS molecules, rather than the LPZ prodrug, is required to acquire plasma and lung tissue concentrations of the active molecule sufficient to kill Mtb [114]. Recently, the use of LPZ has been associated with reduced incidence of TB when compared with the use of omeprazole or pantoprazole [115].

The ATP synthase inhibitor BDQ and Q203 both result in an increase in mycobacterial oxygen consumption rate (OCR) over a range of physiologically relevant oxygen concentrations [35]. It was suggested that bacteria are compelled to utilize cytochrome *bd*-oxidase as the terminal oxidase upon disruption of the flow of electrons through the *bc*_1_-*aa*_3_ pathway by Q203. This postulate is supported by the observation that Q203 completely inhibits respiration in the cytochrome *bd*-oxidase knockout mutant (cydKO), while the Q203 resistant SNP remains unaffected [35]. Furthermore, cydKO is hypersusceptible to Q203 [116,117]. BDQ and Q203 are relatively slow acting drugs with regard to mycobacterial cytotoxicity. Importantly, BDQ both alone or in combination with Q203, show similar slow kill kinetics, but the combination of CFZ with either BDQ or Q203 results in increased OCR and synergistic killing. The triple combination of CFZ, BDQ, and Q203 kills even faster, and results in complete sterility within 5 days. This combination of drugs is far more effective and rapid than the combination of first-line drugs INH and RIF in culture broths and in infected macrophages. BDQ and Q203 push the cell into reductive stress, during which NADH levels increase, and this potentiates the ROS-generating activity of CFZ, leading to mycobacterial killing [35]. These observations suggest that a combination of drugs targeting different components of the respiratory chain may help in the development of more effective therapeutic regimens. Targeting the bacterium at multiple steps of oxidative phosphorylation may be a better strategy for tackling drug-susceptible as well as MDR and XDR-TB. The role of efflux pumps in Mtb drug resistance is well established [118], and therefore, targeting them may help to resensitize Mtb to antibacterial agents. In support of this, verapamil (an efflux pump inhibitor) increases the potency of Q203 [119].

## 4. ATP Synthase

During the transfer of electrons from NADH or succinate to a terminal electron acceptor (such as oxygen), protons are pumped out to generate PMF. ATP synthase can utilize PMF to generate ATP from ADP and inorganic phosphate [120,121]. ATP synthase is highly conserved from bacteria to mammals; it consists of a transmembrane F_o_ complex that is used for ion translocation, and a peripheral F_1_ complex that catalyzes ATP generation. The membrane-embedded F_o_ complex is made up of *a*, *b*, and *c* subunits that are present in a variable stoichiometry of *ab*_2_*c*_8–15_. On the other hand, the hydrophilic F_1_ complex consists of 5 subunits in the stoichiometry α_3_β_3_γδε [122]. The proton movement is utilized by the F_o_ subunit to generate rotation of the oligomeric *c* ring, and this rotation is coupled with the rotation of the attached γ and ε subunits of the F_1_ complex [123,124,125].

During its infection cycle, mycobacteria must survive host-generated stress [126,127]. In order to do so, Mtb utilizes its metabolic flexibility to maintain ATP homeostasis [1,128]. Regulated synthesis of ATP through ATP synthase is critical for the survival of pathogens inside the host. This enzyme is even more important in mycobacterial cells, since they require it for growth on fermentable carbon sources [129]. It must be noted that Mtb downregulates its metabolic rate upon entering macrophages or lung tissue [1,130], and primarily depends on lipids for energy [131]. ATP levels are also reduced in the pathogen upon oxygen and nutrient deficit [9,132]. In the case of *Streptococcus*, ATP synthase is downregulated during biofilm formation [133], which is believed to harbor dormant bacteria. However, the level of ATP in biofilm-resident Mtb is not significantly different to that observed in planktonic bacteria [134]. Also, single cell microscopy has revealed that antibiotic exposure tremendously reduces the ATP levels in *M. smegmatis* cells [135]. ATP synthase in mycobacteria is encoded by the *atpBEFHAGDC* operon (Rv1303–1312). The expression of this operon is tightly regulated to match the cellular ATP requirements, and is therefore closely linked to the respiratory chain activity. This operon is downregulated in hypoxic cultures and in murine lung tissue [8]. Recently, a transcriptional regulator (*blaI*, Rv1846c) of the *atp* operon has been identified [136].

**Figure 3:**
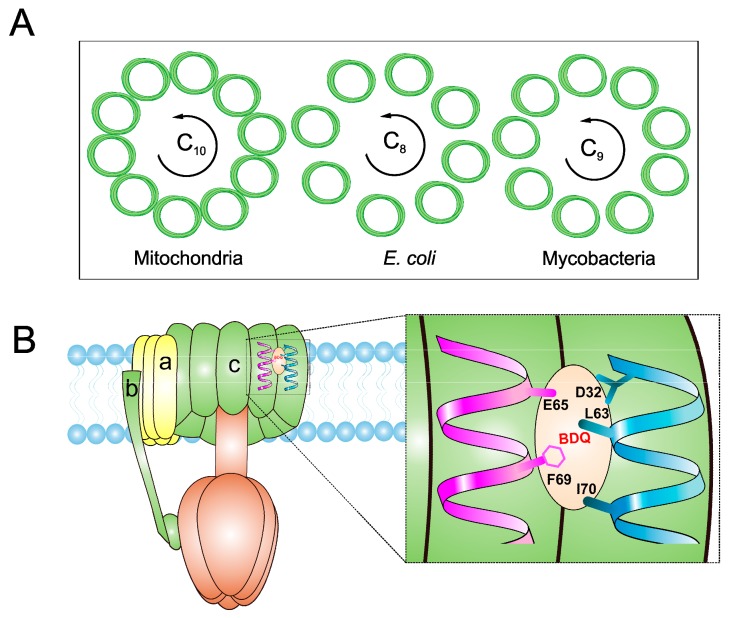
Schematic view of ATP synthase and its interaction with bedaquiline (BDQ). (**A**) Depicts the top view of *C*-ring of ATP synthase F_o_ complex and comparison of *C*-rings of mitochondria (C_10_), *E. coli* (C_8_) and mycobacteria (C_9_). (**B**) BDQ binds between the two c subunits of the *C*-ring. The interaction of BDQ with C ring is illustrated in the zoomed region. BDQ specifically interacts with Glu^65^ (E65), Phe^69^ (F69), Leu^63^ (L63), Asp^32^ (D32), and Ile^70^ (I70) of adjacent c subunits.

The subunit composition of mycobacterial ATP synthase and the mechanism of ATP generation is similar to that of other bacteria, such as *E. coli* and humans (Figure 3A). However, there are subtle differences in Mtb ATP synthase that contribute to its survival in humans [137]. These differences, however, can be exploited for the development of Mtb-specific drugs. These differences include the capability to catalyze the ATP biosynthesis at low PMF of ~100 mV [9] compared with ~200 mV of *E. coli* [138], and the ability to block ATPase activity [139,140] in order to maintain an optimal level of PMF, as seen in many other bacteria [141]. Mycobacterial ATP synthase has several structural differences compared with the human orthologue. This includes a 36 amino acid C-terminal domain extension of subunit α [142], which is not seen in any other prokaryotes or eukaryotes. Interestingly, this extension is critical for the ability of mycobacterial ATP synthase to reduce ATPase activity. The Mtb ATP synthase γ subunit possesses a unique loop (γ^165–178^) of 13 amino acids [143] that also inhibits ATP hydrolysis-driven proton pumping [144]. Interestingly, due to the capability of ATP generation at low PMF, Mtb ATP synthase was expected to have a large *c* ring with more than 10 monomers, as seen in some alkaliphilic bacteria [145,146,147]. However, the crystal structure of the mycobacterial *c* ring revealed that it contains 9 monomers [148]. This is the smallest bacterial *c* ring reported with a H^+^/ATP ratio of 3. 

Given the essentiality of ATP synthase for the survival of mycobacteria on fermentable and non-fermentable carbon sources, and its subtle but peculiar structural differences from the mitochondrial ATP synthase present in humans, this enzyme can be utilized as a potential drug target. However, drugs like oligomycin and *N*,*N*′-dicyclohexylcarbodiimide (DCCD) (that target ATP synthase) are non-selective, and also inhibit generation of ATP in mitochondria and bacteria [149,150], suggesting the conserved nature of this enzyme. Nonetheless, the recent discovery of BDQ as a specific inhibitor of mycobacterial ATP synthase has validated this enzyme complex as a drug target. 

### 4.1. Bedaquiline

Several naturally occurring and synthetic ATP synthase inhibitors (such as DCCD) have been described [151]. However, it was only in 2004 [152,153], that an ATP synthase-targeting lead compound of the diarylquinoline class (R207910) was shown to have potent antimycobacterial activity. Four decades after the development of rifabutin in 1975, a new class of antibiotic that specifically targeted tuberculosis was developed by the Andries group [152]. This compound was later called BDQ, or TMC207, and is sold under the brand name of Sirturo^®^.

Importantly, this compound specifically inhibited the growth of mycobacterial species, and was non-inhibitory to Gram-positive bacteria, such as *Nocardia*, and Gram-negative bacteria such as *E. coli*. More importantly, this compound was able to kill MDR and XDR Mtb strains with MICs in the range of 0.03–0.12 µg/mL. Interestingly, usage of 10× MIC for 12 days resulted in a 3-log reduction of bacterial colony forming units (cfu). This killing was time dependent, rather than being concentration dependent. The delayed onset of killing could be explained by the metabolic remodeling of mycobacterial cells upon BDQ exposure. This includes upregulation of the dormancy regulon, ATP synthase, isocitrate lyase, and cytochrome *bd* oxidase. Additionally, there was a downregulation of DNA/protein biosynthesis and efflux pump expression, in order to conserve energy. The drug also demonstrated significant in vivo activity in a murine model of TB infection. Importantly, its use alone led to a similar reduction of mycobacterial load in the lungs as that of a combination of frontline drugs (RIF + INH + pyrazinamide (PZA)). Moreover, its use in combination with these drugs resulted in a 2-log greater reduction in mycobacterial load compared to the “no BDQ” group. Importantly, BDQ also showed desirable pharmacokinetic properties [152]. Furthermore, use of the Wayne model demonstrated that BDQ efficiently targets both replicating and non-replicating mycobacteria [9]. The drug is posited to be more effective against non-replicating bacteria than those that are actively replicating; this could be due to 5–10-fold lower ATP levels in the non-replicating dormant bacteria [154]. The ability to kill non-replicating bacteria correlates with BDQ’s exceptionally high bactericidal activity in mouse TB models [155,156,157], and also with the reduced time for sputum culture conversion in TB patients [158,159].

In an effort to identify the target of BDQ, whole genome sequencing was performed. Mutations in *atpE*, which codes for the F_o_ subunit of ATP synthase, were associated with drug resistance [152]. In another target identification effort, mycobacterial membrane extract was passed through a column of BDQ-coated Sepharose beads. This approach also identified subunits of ATP synthase. These experiments were followed by surface plasmon resonance assays, which demonstrated that atpE binds to a BDQ analogue with a K_d_ of 500 nM [160]. The MIC_50_ in the case of human mitochondrial ATP synthase was more than 20,000-fold higher than that of the bacterial enzyme, underscoring the high degree of selectivity afforded by BDQ [161]. A high-resolution crystal structure of mycobacterial ATP synthase, with and without BDQ, has provided insights into the mechanism of BDQ binding, and will also help in structure-based drug design [148]. It was suggested that one or more BDQ molecules could specifically associate with ion binding site of ATP synthase in the c ring of the F_o_ rotor. BDQ interacts with ~135 Å^2^ of the c ring of the F_o_ rotor consisting of nine residues (Gly62, Leu63, Glu65, Ala66, Ala67, Tyr68, Phe69, Ile70, and Leu72) through van der Waals interactions (Figure 3B). BDQ’s dimethylamino moiety extends into the ion-binding site of C-rings. The interaction is then stabilized by an intermolecular H-bond between the dimethylamino group and Glu65 [148]. BDQ has also been tested against NTM, *M. abscessus*, which affects cystic fibrosis patients [162], and against *M. avium* and *M. intracellulare* [163]. Additionally, it was found to be active against *M. abscessus* in a zebrafish model of infection [164]. Furthermore, BDQ was effective against *M. leprae* [165], but bacteriostatic in the case of *M. avium* [166], and inactive in *M. abscessus* mouse infection [167,168].

Moreover, BDQ, when combined with other TB drugs, helped to reduce the time of treatment [156,157,169]. A synergistic effect of BDQ and PZA was seen in a mouse model of TB [170]. BDQ was also synergistic with the cell wall synthesis inhibitor, SQ109, as it improved the killing rate and lowered the MIC [171]. Another cell wall synthesis targeting compound, BTZ043, was also found to be synergistic with BDQ in vitro [172]. BTZ043 is a benzothiazinone which inhibits DprE1, an enzyme involved in arabinan biosynthesis [173]. The increased efficacy of BDQ, when used in combination with cell wall biosynthesis inhibitors, could be due to its increased penetration. Furthermore, clinical trials are ongoing for the combination therapy of BDQ with another recently approved drug, delamanid [174]. BDQ results in prolonged QT interval, however, co-administration of RIFor rifapentine with BDQ increases its clearance, likely due to their activity as CYP3A4 inducers, which is involved in BDQ metabolism [175]. Thus, we infer that the currently approved BDQ–RIF combination therapy should be re-evaluated. Furthermore, the addition of ketoconazole, a CYP3A4 inhibitor with BDQ increased the QT interval [176]. A study conducted in human hepatocytes to understand the BDQ metabolism identified *N*-dealkylation, a novel metabolic pathway. In this study, CYP3A4, CYP2C8, and CYP2C19 were shown to be involved in BDQ *N-*demethylation [177]. This kind of study is important to understand and prevent BDQ associated adverse drug reactions.

The rate of generation of BDQ-resistant mutants is one in 10^8^ bacteria. Certain mycobacteria, including *M. novocastrense*, *M. xenopi*, and *M. shimoidei*, are naturally resistant to BDQ, due to polymorphisms at the *atpE* locus [168,178]. BDQ resistance is also acquired, and is present in bacteria with cross-resistance to CFZ. This cross-resistance is due to mutations in Rv0678, a transcriptional repressor of the MmpL5 efflux pump [179,180,181]. BDQ is very effective against drug-susceptible MDR and XDR-TB; however, due to its toxicity (attributed to the increased QT interval) and the development of BDQ-resistant strains [182], its use is currently limited to MDR and XDR-TB patients. 

### 4.2. Squaramides

After the discovery of BDQ, several studies have been conducted in order to identify novel molecules that target the mycobacterial oxidative phosphorylation pathway [100,101,103,105]. In one example, AstraZeneca employed a luminescence-based method in which inverted membrane vesicles from mycobacteria were charged with NADH and formation of ATP was monitored. Following HTS of 900,000 compounds, two lead classes, namely squaramide and imidazo [1, 2-a] pyridine ethers (IPE), were identified. Given their poor solubility, pharmacokinetics, and bacteriostatic nature, IPE were deprioritized, and squaramides were further developed [183]. This resulted in the identification of 31f, which exhibited potent antituberculosis activity (IC_50_ of 0.03 µM in proliferation assays). Similar to the case with BDQ, docking studies and the generation of spontaneous mutants identified ATP synthase as the target of squaramide. However, the mode of binding employed by squaramides is different to that of BDQ. 31f is predicted to bind to the interface of ATP synthase subunits *a* and *c*. This prediction was supported by the observation that the K179N mutation of subunit *a* and the D28N mutation of subunit *c* of ATP synthase engendered resistance to 31f. Importantly, squaramides were also found to be active against single drug resistant clinical strains, including BDQ. Furthermore, squaramides show a similar MIC for BDQ-resistant mutant strains and do not show cross-resistance, suggesting that they interact with a different binding site. Lead compound 31f was also effective in a mouse model of acute TB infection. Further studies are required to see whether squaramides can potentiate the activity of current TB drugs, and whether they will be effective in a chronic TB infection model.

## 5. Uncouplers of Proton Motive Force: Pyrazinamide and SQ109

PMF generated by ETC plays a critical role in bacterial growth and survival. During hypoxic growth, dissipation of PMF by uncouplers, such as valinomycin and nigericin, can cause death in the hypoxic cultures [9]. The importance of PMF as a drug target is further validated by the observation that efflux of a number of drugs from mycobacterial cells is dependent on PMF [184]. However, general uncouplers of PMF are not sufficiently selective to be used as antimycobacterial agents, and the development of specific PMF uncouplers remains an area of interest. Importantly, PZA, which is currently being used as a first-line anti-TB drug along with RIF and INH, can disrupt the PMF in mycobacterial cells. Another lead compound, SQ109, also targets the PMF and oxidative phosphorylation [185]. 

PZA was discovered in the early 1940s, and is effective in mouse models of TB [186] and in TB patients [187]. Consequently, the drug is used as a frontline agent to treat TB. PZA is a prodrug that requires acidic conditions [188] in order to be hydrolyzed by pyrazinamidase or nicotinamidase to pyrazinoic acid (POA), the active form of the drug [189]. PZA-resistant mutants often have variations in the pyrazinamidase gene, which is encoded by *pcnA* [190,191,192]. PZA is a multitarget drug that dissipates the PMF, inhibits ATP synthesis [193], inhibits membrane transport [194], and also reduces the activity of other proteins [195]. The first clue that PZA could dissipate PMF was the requirement of acidic pH for its bactericidal activity [196]. PZA requires 4–5 fold higher concentration (850 μg/mL) at pH 6.5 compared to pH 5.5 (200 μg/mL) [194]. The active form of the drug, POA, is a weak acid that accumulates inside the cell in acidic conditions [196]. This weakly acidic property helps POA (and the structurally unrelated benzoic acid) to disrupt the membrane potential of Mtb under acidic conditions [194]. In line with this observation, PZA is more active against non-replicating dormant bacteria, as compared to actively replicating bacteria [197] and old cultures [198]. These findings are further supported by the fact that PMF uncouplers synergize with PZA in order to deplete ATP depletion and enhance mycobacterial killing [194]. However, the exact mechanism by which PZA dissipates the membrane potential remains unknown.

SQ109 is structurally related to the frontline TB drug ethambutol, and was developed by Sequella Incorporation in collaboration with Laboratory of Host Defenses, NIH. It has successfully completed phase 2 clinical trials, and is active against drug-susceptible MDR and XDR-TB [199]. Although in TB patients, SQ109 monotherapy for 14 days did not reduce bacterial burden [200], a recent phase 2b-3 clinical study suggested an 80% increase in the culture conversion rate by addition of SQ109 to the standard regimen [201]. Initially, SQ109 was reported to interfere with the assembly of mycolic acid in the mycobacterial cell wall. This was attributed to its ability to target MmpL3, a membrane transporter involved in uptake of trehalose monomycolate [202,203]. However, SQ109 is also active against other organisms, including *Helicobacter pylori* [204], *Trypanosoma cruzi* [205], *Neisseria gonorrhoeae*, *Candida albicans* [206], and *P. falciparum* [185]. Since these organisms lack a functional homolog of MmpL3, it is highly likely that MmpL3 is not the primary target of SQ109. This hypothesis is further supported by the absence of spontaneously resistant mutants, a phenomenon observed with many drugs that inhibit multiple targets. Recently, Li et al., have demonstrated that SQ109 and its analogues inhibit MK biosynthesis, and interfere with respiration and generation of PMF [185,205]. It acts as an uncoupler, collapsing both ΔpH and Δψ, leading to a decrease in ATP synthesis [185]. Importantly, SQ109 synergizes with BDQ and CFZ, further suggesting that it affects oxidative phosphorylation [206]. Further studies are required to completely understand the complex mechanism by which SQ109 inhibits mycobacterial cell growth.

## 6. Conclusions

**Figure 4:**
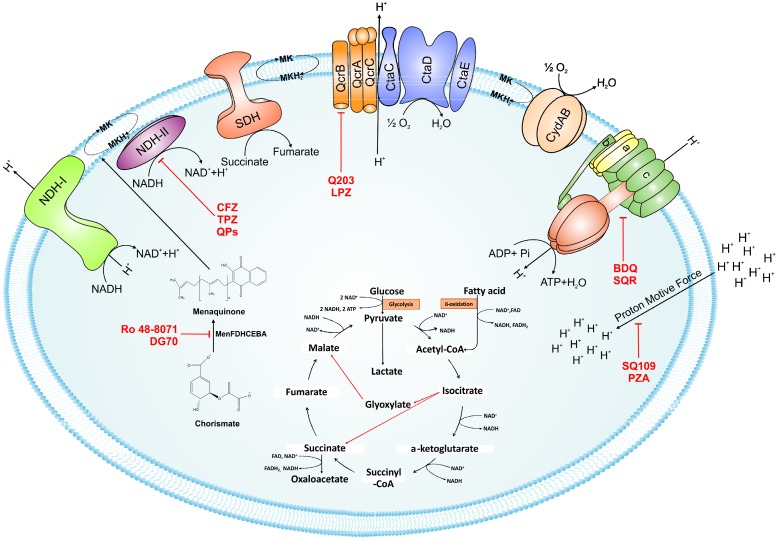
Schematic representation of the mycobacterial electron transport chain and its inhibitors. NADH derived via glycolysis and tricarboxylic acid (TCA) cycle feds electrons into the electron transport chain by NADH dehydrogenase. The menaquinone (MK) pool can be reduced by primary dehydrogenases such as NADH dehydrogenases (NDH1 and NDH2) and via succinate dehydrogenase (SDH). Electrons from the menaquinone pool are accepted directly by cytochrome *bd*-type terminal oxidase or via *bc*_1_-*aa*_3_ supercomplex. A proton motive force (PMF) is generated during electron transport chain because of pumping of protons across the membrane. This PMF is used by the ATP synthase to generate ATP. Drugs that target the oxidative phosphorylation are shown by red flathead arrows. Abbreviations: CFZ, clofazimine; TPZ, trifluoperazine; QPs, quinolinyl pyrimidines; Q203, imidazopyridine amide; LPZ, lansoprazole; Ro 48-8071, (4-bromophenyl)[2-fluoro-4-[[6-(methyl-2-propenylamino)hexyl]oxy]phenyl]-methanone; DG70,biphenyl amide; BDQ, bedaquiline; SQR, squaramide; SQ109, *N*-adamantan-2-yl-*N*-((E)-3,7-dimethyl-octa-2,6-dienyl)-ethane-1,2-diamine; PYZ, pyrazinamide. Red arrows in the TCA cycle depict the glyoxylate shunt.

Despite the presence of alternate respiratory complexes, oxidative phosphorylation is an excellent target for the development of new antimycobacterial drugs (Figure 4). This statement is validated by the presence of a number of its inhibitors in the current TB drug pipeline. Most notable among these inhibitors are Q203 (an inhibitor of *bc*_1_-*aa*_3_ complex) that has entered clinical trials, and BDQ (ATP synthase inhibitor) that has been approved for treatment of MDR TB. It is worth noting that inhibitors of other components of oxidative phosphorylation, such TPZ&QPs (NDH-2 inhibitor), Ro 48-8071 & DG70 (inhibitors of MK biosynthesis) and SQ109 (PMF disruptor) are potent blockers of mycobacterial growth, in vitro and in vivo. We believe that co-inhibition of parallel respiratory components could significantly shorten the duration of TB treatment. This belief is supported by the enhanced susceptibility of *cydAB* deletion mutant to BDQ or accelerated killing of Mtb cells by the combination of CFZ with BDQ or Q203. Such combinations shall be tested, in vivo, to further test this hypothesis. 

## Competing Financial Interests

The authors declare that they do not have any competing financial interests. 

## Figures and Tables

**Table 1:** Summary of potential drug molecules with their respective targets, bactericidal properties, and structures.

Drug	Target	Properties	Structure
Thioridazine	NDH-2	MIC_90_-8–15 µg/mL Approved	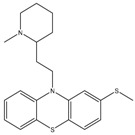
Trifluoperazine	NDH-2	MIC_90_-19.2 µg/mL	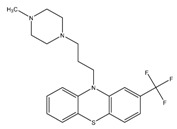
Clofazimine	NDH-2	MIC_90_-0.25 µg/mL Phase III clinical trials	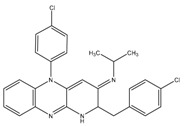
Quinolinyl pyrimidine	NDH-2	MIC_90_-8-32 µg/mL	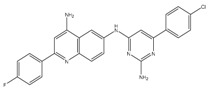
3-Nitropropionate (3NP)	SDH	MIC_90_-3.3 µM Active in vivo	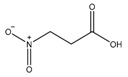
DG70	MenG	MIC_90_-4.8 µg/mL	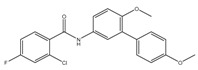
Aurachin RE	MenA	MIC_90_ < 12.5 µg/mL	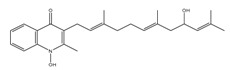
Ro 48-8071	MenA	MIC_90_-3 µg/mL	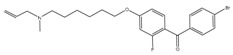
7-Methoxy-2-naphthol	MenA	MIC_90_-3 µg/mL	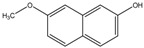
Allylaminomethanone	MenA	MIC_90_-1 µg/mL	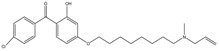
Aurachin D	Cytochrome bd oxidase	MIC_90_-85 µM	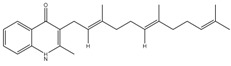
PABS (Phenoxyalkylbenzimidazoles)	QcrB	MIC_90_-0.056 µM	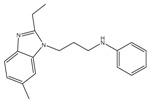
Q203 (imidazopyridine amide)	QcrB	MIC_50_-0.28 nM ex vivo MIC_50_-2.7 nM in vitro Phase I clinical trials	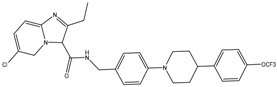
Pyrrolo[3,4-c]pyridine-1,3-dione	QcrB		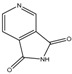
Lansoprazole	QcrB	MIC_90_-104 µg/mL Active in vivo	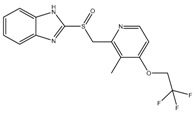
Bedaquiline	ATP synthase	MIC_90_-0.004-0.13 µg/mL Approved for MDR-TB Phase III clinical trials for DS-TB	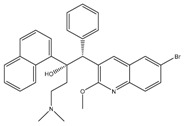
Squaramide	ATP synthase	MIC_90_-0.5 µM Active in vivo Pre-clinical trials	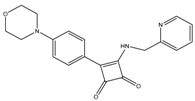
SQ109	PMF	MIC_90_-0.78 µg/mL Phase II clinical trials	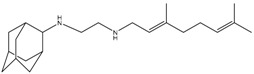
Pyrazinamide	PMF	MIC_90_-100 µg/mL Approved	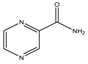

## Abbreviations

3NP	3-Nitropropionate
ADP	Adenosine diphosphate
ATP	Adenosine triphosphate
BCG	Bacillus Calmette Guerin
BDQ	Bedaquiline
cfu	Colony forming units
CFZ	Clofazimine
CO_2_	Carbon dioxide
cydKO	Cytochrome bd-oxidase knockout mutant
DCCD	*N*,*N*′-dicyclohexylcarbodiimide
DprE1	Decaprenylphosphoryl-D-ribose oxidase
ETC	Electron transport chain
FADH_2_	Reduced flavin adenine dinucleotide
FRD	Fumarate reductase
H^+^	Proton
H_2_O	Water
HIV	Human immunodeficiency virus
HTS	High throughput screening
INH	Isoniazid
IP	Imidazo[1,2-a]pyridines
LPZ	Lansoprazole
LPZS	Lansoprazole sulphide
MDR	Multi drug resistant
MIC	Minimum inhibitory concentration
MK	Menaquinone
MKH_2_	Menaquinol
Mtb	Mycobacterium tuberculosis
NAD^+^	Nicotinamide adenine dinucleotide
NADH	Reduced nicotinamide adenine dinucleotide
NDH-1	Type I NADH dehydrogenase
NDH-2	Type II NADH dehydrogenase
NQR	Sodium-pumping NADH dehydrogenase
NTMs	Non-tuberculous mycobacteria
OCR	Oxygen consumption rate
OSB	*O*-succinyl-1-benzoate
PAB	Phenoxyalkylbenzimidazole
Pi	Inorganic phosphate
PMF	Proton motive force
POA	Pyrazinoic acid
PZA	Pyrazinamide
Q	Ubiquinone
RIF	Rifampicin
ROS	Reactive oxygen species
SAR	Structure–activity relationship
SDH	Succinate dehydrogenase
SNP	Single nucleotide polymorphism
TB	Tuberculosis
TCA cycle	Tricarboxylic acid cycle
TPZ	Trifluoperazine
TZ	Thioridazine
XDR	Extremely drug resistant
